# Lexibank 2: pre-computed features for large-scale lexical data

**DOI:** 10.12688/openreseurope.20216.2

**Published:** 2025-06-23

**Authors:** Frederic Blum, Carlos Barrientos, Johannes Englisch, Robert Forkel, Simon J. Greenhill, Christoph Rzymski, Johann-Mattis List

**Affiliations:** 1 Department of Linguistic and Cultural Evolution, Max-Planck-Institute for Evolutionary Anthropology, Leipzig, Saxony, 04103, Germany; 2 Chair for Multilingual Computational Linguistics, Universitat Passau, Passau, Bavaria, Germany; 3 School of Biological Sciences, The University of Auckland, Auckland, Auckland, New Zealand

**Keywords:** cross-linguistic data, historical linguistics, computational linguistics, lexibank, comparative wordlists

## Abstract

Large-scale lexical and grammatical datasets nowadays play an important role in comparative linguistics. However, the lack of standardization remains a challenge exacerbating extension and reuse of published data. We present an updated version of Lexibank, a large-scale lexical dataset, expanding on previous efforts to standardize and unify cross-linguistic data. This new version includes over 3,100 languages and more than one-and-a-half million word forms, substantially broadening the scope and utility of the previous resource. Our dataset has been systematically curated using a dedicated computer-assisted workflow designed specifically for the lifting of published wordlist data to the standards recommended by the Cross-Linguistic Data Formats initiative. The expanded dataset features standardized references to language varieties, standardized semantic glosses that reference the concepts expressed by individual word forms, and standardized phonetic transcriptions for all word forms that our repository contains. Based on those standardizations we pre-compute semantic and phonological features, which can be used to carry out extensive automated analyses. We illustrate this potential by providing dedicated database queries to (1) infer words that are similar in pronunciation and meaning, (2) identify concepts that are colexified across languages in our sample, and (3) assess the semantic diversity of etymologically related words. These queries are not only fast to execute but also global in their scope, due to the largescale coverage provided by Lexibank 2. The queries are also easy to extend, thus having the potential to contribute to various studies in historical linguistics, linguistic typology, and related disciplines. The updated dataset is a substantial step forward in the effort to create comprehensive, standardized, and accessible linguistic resources.

## Introduction

Large-scale lexical and grammatical datasets have become indispensable tools in comparative linguistics, offering researchers the ability to analyze and compare languages in new ways. Grammatical databases have been used to address questions on linguistic complexity ( Shcherbakova *et al.*, 2023; Skirgård *et al.*, 2023), collections of corpus data have been used to study lengthening effects in speech across languages ( Blum *et al.*, 2024c), and lexical wordlists have helped to shed light on cross-linguistics aspects of lexical semantics and human cognition ( Jackson *et al.*, 2019; Tjuka, 2024). As the field of linguistics continues to make use of digital resources, the demand for standardized and accessible datasets has grown substantially. However, the diversity of data formats and the lack of consistent methodologies across various linguistic datasets remain significant challenges, limiting the full potential of these resources. At the same time, more and more data became available, opening up further challenges with respect to re-use and validation of existing datasets.

One of the primary obstacles for the creation of large-scale linguistic datasets is the lack of standardization in individual resources. Language names used to identify language varieties are often ambiguous and may easily change over time. Semantic glosses often differ greatly across datasets, even if they are intended to elicit the same concepts denoted by individual word forms. Phonetic transcriptions that would describe the pronunciations of word forms are often either lacking completely or they follow idiosyncratic systems that cannot be directly interpreted without further information. When datasets lack uniformity in format, transcription, and structure, it becomes difficult to conduct reliable cross-linguistic studies, drastically restricting the integration and re-use of data from different sources. As a result, the full potential of linguistic data can be rarely employed in cross-linguistic studies, despite the fact that the raw amount of such data has been drastically increasing over the past decades.

The major idea of the Lexibank repository ( https://lexibank.clld.org, List *et al.*, 2022) was to address this problem by consistently standardizing as many lexical datasets as possible and providing access to the datasets within a unique repository. When Lexibank was launched for the first time in 2022, the repository consisted of 100 individual datasets from which phonological and lexical features were automatically computed ( List *et al.*, 2022). The data in this version of Lexibank (v0.2) thus consisted mainly of *structural features* for a larger number of language varieties that were derived from the underlying data using an automated workflow. Later in 2023, the Lexibank repository was adjusted by providing not only the computed features, but also a selection of the largest wordlists available for each language ( List *et al.*, 2023). In this version (v1.0), the wordlists of 74 datasets were provided using Cross-Linguistic Data Formats (CLDF, https://cldf.clld.org, Forkel *et al.*, 2018) to standardize major aspects of lexical data, most importantly the representation of language, meaning, and form. Thanks to the standardization, it was possible to supplement the improved Lexibank version with a CLLD web-application ( Forkel, 2014) that offers users convenient ways to browse the data aggregated by Lexibank interactively ( https://lexibank.clld.org).

Lexibank builds on CLDF to unify cross-linguistic lexical data along the three basic dimensions of *language*, *concept*, and *form* (for details on these three dimensions in historical linguistics, see List, 2016). In order to ensure that lexical data can be easily compared along these dimensions, Lexibank follows CLDF in using *reference catalogs*. Reference catalogs are large collections of metadata referencing major scientific constructs. While conventions to refer to scientific constructs, such as names for languages, semantic glosses for concepts, or transcriptions for speech sounds, often vary across datasets, traditions, and times, reference catalogs provide a unique anchor point with stable identifiers that allows scholars to reference the constructs consistently. Lexibank uses Glottolog ( https://glottolog.org, Hammarström *et al.*, 2024) and its Glottocodes ( Forkel & Hammarström, 2022) as a basic reference for languages. For the handling of semantic glosses that refer to individual concepts, the Concepticon catalog is used ( https://concepticon.clld.org, List *et al.*, 2025), linking semantic glosses employed in several hundred published concept lists to unique *Concept Sets* ( List *et al.*, 2016a). To unify phonetic transcriptions across individual cross-linguistic lexical datasets, the standard transcription defined by the Cross-Linguistic Transcription Systems initiative (CLTS, https://clts.clld.org, List *et al.*, 2024) is used. CLTS links several thousand speech sounds that are represented by a fixed set of feature values across different transcription systems, thereby offering a refined version of the International Phonetic Alphabet (IPA, IPA, 1999) that resolves several ambiguities ( Anderson *et al.*, 2018).

Lexibank aggregates wordlist data from a collection of individually compiled CLDF datasets that were all assembled with the help of the *Lexibank workflow*. This workflow builds on CLDFBench ( Forkel & List, 2020), a Python package that facilitates the conversion of individual data to CLDF. The Lexibank workflow uses the PyLexibank package ( Forkel *et al.*, 2025a), built on top of CLDFBench, to convert lexical data (that may be provided in various idiosyncratic formats) into standardized CLDF datasets. Those datasets link languages to Glottolog, map concepts to Concepticon, and represent phonetic transcriptions in accordance with CLTS ( List *et al.*, 2022).

Once the cross-linguistic data has been assembled in standardized form, it is straightforward to extract several features that provide information on certain properties on individual language varieties. Here, Lexibank uses CL Toolkit ( https://pypi.org/project/cltoolkit), a software package that extracts phoneme inventory information as well as dedicated phonological and lexical features (syllable complexity, full and partial colexifications, see List, 2023a) from wordlists accessible in CLDF. All in all, this results in a collection of 30 phonological features, 30 lexical features, and detailed information on phoneme inventory sizes for several thousand languages.

In this study, we present Lexibank 2, a new version of Lexibank that comes with several improvements in comparison to earlier versions. Lexibank 2 now includes data from over 3,100 languages with more than one-and-a-half million different word forms, adding data from more than 1,000 new languages and over 1,000,000 new lexical forms to the collection. The dataset has been systematically expanded and enhanced using an improved data curation workflow that addresses several shortcomings of the previous Lexibank version. In addition, we now enrich the dataset systematically by integrating it with pre-computed features that allow for an improved handling of phonetic and semantic similarities between words across and within the same language. We illustrate the usefulness of these new features by providing fast and efficient queries that allow us to explore the data in various ways, opening up new possibilities for linguistic research. By providing a comprehensive and comparable resource, it enables automated inferences of phonological and lexical features across languages, facilitating large-scale comparative studies. As a freely available resource, Lexibank aims to support and stimulate further studies into the diversity of human language through the combination of digital tools and collaborative efforts.

## Materials and methods

### Datasets

Lexibank 2 consists of 134 individual CLDF datasets, covering 3,107 languages from 207 language families with a total of 1,734,794 word forms. All datasets have been standardized by linking languages to Glottolog ( Hammarström *et al.*, 2024, Version 5.1), mapping concepts to Concepticon ( List *et al.*, 2025, Version 3.4.0), and using CLTS ( List *et al.*, 2024, Version 2.3) as the basis for phonetic transcriptions. Similar to previous versions of Lexibank, the preparation of the data featured in Lexibank 2 was based on collaborative efforts. Individual teams coordinated the different tasks involved in the Lexibank workflow for CLDF conversion, making sure that concept lists were properly linked to Concepticon by extending the Concepticon reference catalog ( Tjuka *et al.*, 2023), double-checking the assignment of languages to Glottocodes, and discussing open questions in the creation of orthography profiles ( Moran & Cysouw, 2018), which are needed to convert original transcriptions to standardized CLTS representations. Additional corrections included the removal of duplicate entries that resulted from individual conversion errors in individual datasets, the adjustment of the citation information, and the correction of unnatural phonetic transcriptions that we detected by investigating consonant clusters in the data.

The full table of datasets is provided in Table 7, Appendix A. When preparing the data to be included in Lexibank 2, all datasets were thoroughly checked by us in teams, paying particular attention to the phonetic transcriptions, the mapping to Glottolog, and the linking of the concepts to Concepticon. After all these checks had been carried out and new versions of our three reference catalogs (Glottolog, Concepticon, and CLTS) had been integrated, new releases of the data were carried out and archived with Zenodo. From these releases – which we store along with additional bibliographic information in a separate table – the Lexibank 2 data was then aggregated automatically. The updated version of Lexibank improves on the previous release by further increasing data quality. For example, all datasets now have their concept list mapped to Concepticon ( List *et al.*, 2025). This makes it easier to compare the data across individual datasets, with benefits that we will highlight in the next section. All new datasets have been compiled with individual orthography profiles ( Moran & Cysouw, 2018), mapping the graphemes from the original source to the base transcription systems underlying CLTS. Datasets for which this was not feasible have been excluded from the release. Individual datasets, like the Intercontinental Dictionary Series, received major updates for subsets of the data ( Miller & List, 2024), which have been incorporated into Lexibank as well. The improved data quality criteria ensure that Lexibank maintains high quality standards, while making data from different studies reusable.

### Data collections

The datasets and language varieties in Lexibank 2 are assigned to one or more *collections*, depending on the basic information they convey. A collection is a subset of wordlists in the repository that conforms to a specific criterion. One and the same wordlist for one and the same language variety can be assigned to different collections at the same time. Figure 2 shows the criteria to assign individual datasets to collections. In order to be included into the Lexibank repository, a dataset must be available in the form of a CLDF wordlist in which concepts have been mapped to Concepticon, languages are linked to Glottolog, and phonetic transcriptions follow the CLTS transcription system. The L exiC ore collection (constituting the basis of all data in Lexibank) consists of all language varieties that have at least 80 different concepts mapped to Concepticon. This assures a minimum amount of coverage for comparative studies and provides the basis for the automated computation of sound inventories and phonological features that was introduced in Lexibank 0.2 ( List *et al.*, 2022). Similarly, C licsC ore consists of wordlists for individual languages with at least 180 different concepts mapped to Concepticon. This collection is used for the automated computation of lexical features, including individual full colexifications ( Rzymski *et al.*, 2020) and partial colexifications ( List, 2023a). Datasets and language varieties assigned to the C ogC ore collection include manually identified cognate sets that show which words are etymologically related (for an extended reuse of this subset, see Häuser & List, 2025). The P rotoC ore collection builds upon C ogC ore and includes only those cases where additionally proto-forms have been reconstructed.

**Figure 1.**
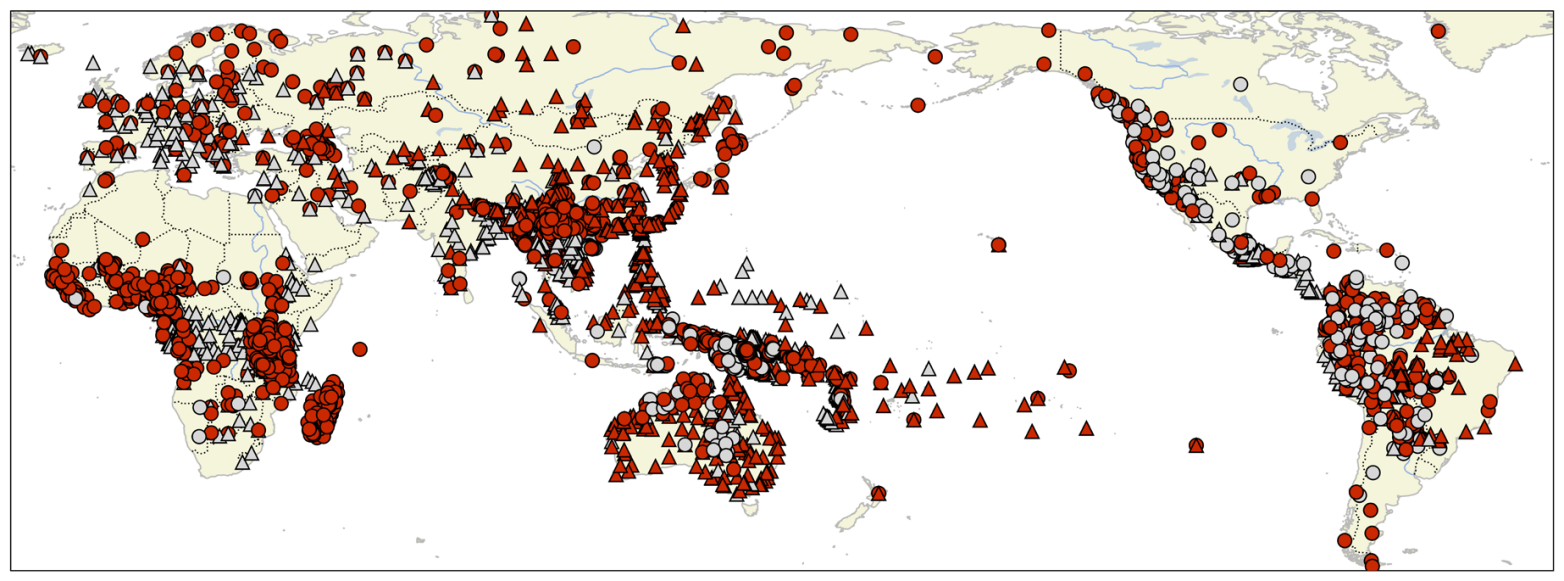
Map showing the global distribution of languages attested in Lexibank 2.

The assignment of languages to collections is done at the level of the datasets but adds – where possible – a computational component. All datasets that were selected for the inclusion in Lexibank 2 were assigned to one or more subsets before aggregation. When aggregating the data, however, those properties that can be inferred automatically (e.g. the number of mappings to distinct Concept Sets in Concepticon), may override the decisions provided prior to aggregation at the level of the datasets. Figure 1 presents a map with all languages in the data with information about the membership of each language in the basic collections.

### Workflow for data curation

With Lexibank 2, we have now not only increased the number of datasets from 76 to 134, we have also improved the selection of datasets and the criteria to select datasets for the inclusion in Lexibank. In addition, we have further enhanced the aggregation workflow, modifying the practice of selecting only data from specific language varieties. Instead, we now aggregate the data from all datasets while at the same time providing interested users with the possibility to select only a balanced subset of the data in which every language variety is only represented by a single wordlist taken from one dataset. In the following, we will briefly discuss these three major improvements made to the new version of the repository.

***Phonetic transcription as the norm.*** As a first improvement, Lexibank no longer offers datasets that do *not* provide standardized phonetic transcriptions. All 134 datasets are available in phonetically transcribed form, with the transcriptions being individually produced with the help of the workflows that were explained in detail in List *et al.* (2022). In order to enhance the previous Lexibank collection, which had still included several collections of wordlists lacking phonetic transcriptions entirely, we followed a two-fold strategy, according to which we deliberately removed certain datasets that lack phonetic transcriptions from the Lexibank collection, while at the same time investing additional time to compute phonetic transcriptions for several datasets that would only provide orthographic forms before ( Hantgan & List, 2023; List, 2023a; Miller & List, 2024). In order to ease the curation of individual datasets, we added new routines dedicated to checking the sanity of the transcriptions in the Lexibank datasets by extending the PyLexibank package ( Forkel *et al.*, 2025a). These include detailed commands allowing us to check the conversion process of graphemes to CLTS-conform segments, as well as a specific command that infers large consonant clusters from the transcription data in order to point to potential problems in the transcription process (see List, 2018 for an early example on consonant cluster inference).

***Improved workflow for data aggregation.*** While individual word forms from selected languages were already introduced with Lexibank 1, this version did not provide access to all datasets upon which the repository was originally built. Since Lexibank aggregates data from different sources, there are many cases in which information on the same language can be obtained from multiple datasets that differ regarding the size of the data they cover and in the individual concepts for which they provide translations. When aggregating data for Lexibank 1, the aggregation workflow would select only one dataset per language variety, using the number of word forms as the sole selection criterion. All alternative sources would be disregarded and excluded from both the comparative word-list and the web application. The aggregation workflow in Lexibank 2 introduces an alternative aggregation technique. Instead of deliberately excluding datasets from the repository, we now retain all data, while at the same time flagging those datasets that provide the largest number of words for a given language variety. This allows users to work both with *all* data available in Lexibank, while at the same time being able to select only one representative per language – where needed. We consider the variety of each glottocode as the most representative which has the most distinct concept mapped to Concepticon. Those varieties are tagged as part of the *Selexion* collection. As an additional improvement, the new workflow for data aggregation now assigns sources at the level of all individual word forms, thus allowing users to trace each datapoint to the original source from which it was taken.

***Pre-computing phonetic and semantic features.*** As a third improvement, we expand the data with pre-computed features that systematically integrate information from the reference catalogs that are linked in the individual source datasets. In contrast to the previous abstract features included in Lexibank, the phonetic and semantic features allow us to compare and analyze the individual word forms. With respect to phonetic features, we systematically convert the segmental representation of word forms in Lexibank to two different sound class representations. Sound classes have been originally proposed by Dolgopolsky (1964), who lumped all consonants into ten major classes, assuming that correspondences within classes are more likely to occur inside than across classes (see List, 2014b for further details). In several computational approaches to cognate detection, scholars use Dolgopolsky’s consonant classes to identify words that are potentially related by assigning all words that match in their first two consonant classes to the same cognate set ( Turchin *et al.*, 2010). This approach has also successfully been used for large-scale language classification Blum *et al.* (2025b). In addition to representing all phonetic transcriptions by their Dolgopolsky sound classes, Lexibank 2 also provides the sound class model proposed by List (2014b). Both sound class representations in Lexibank 2 are retrieved from the CLTS reference catalog ( List *et al.*, 2024).

With respect to semantic features, we employ the colexification network by Rzymski *et al.* (2020) to assign all concepts that gloss the word forms in Lexibank 2 to their *central concept*. The central concept here refers to the concept that contains most links to other concepts within the community to which it was assigned during the community analysis carried out in the creation of the CLICS ^3^ database. The central concepts allow us to determine in a straightforward way whether two words recur in the same community in colexification analyses and thus provides a very rudimentary measure for semantic similarity. In addition, all concepts are tagged with respect to the most common basic vocabulary lists in which they occur. This allows us to filter individual analyses in such a way that only basis concepts are taken into account. Information on central concepts and on the basic concept lists in which concepts occur are retrieved from the Concepticon reference catalog ( List *et al.*, 2025). Since the usage of pre-computed features is still rather experimental at this stage, we restrict the number of features provided to the ones mentioned here. In the future, however, the amount of pre-computed features can be easily extended further.

### Queries for data exploration

In 2020, Bernard Comrie approached us asking if we could help to compare individual word forms in one language against the whole of the data in Lexibank (which had by then not yet been officially published). Inspired by this idea, we began to test different search methods over the past years, trying to offer some kind of a service that would make it easy for scholars interested in particular aspects of Lexibank data to obtain quick results in order to start with a more thorough data exploration afterwards. These queries were never meant to be the endpoint of analysis, but rather as a convenient starting point for *exploratory data analysis* ( Morrison, 2014), helping scholars working with cross-linguistic data to search for interesting patterns before initiating a more targeted and more thorough investigation. Later, this developed into the idea to extend queries to a set of *services*, offering a functionality similar to the BLAST tool in evolutionary biology ( Altschul *et al.*, 1990). While BLAST itself uses sophisticated methods for automated sequence comparison to assemble its matches, the basic fuel that powers our query engine are the pre-computed phonetic and semantic features, as they allow us to identify phonetically and semantically similar words in a very fast way. Still far away from the power and possibilities of BLAST – also resulting from the fact that linguistic data and processes differ from data in bioinformatics ( Blum & List, 2023; List, 2023b; List *et al.*, 2016b) – we consider the queries that we discuss in detail in the following as a first and promising attempt that could turn into a dedicated *Basic Linguistic Search Service* (BLISS) that runs on the top of Lexibank 2.

When experimenting with different ways to query the data in Lexibank, we realized that the most efficient way to deal with large CLDF datasets is to convert the tabular files in CLDF to an SQLite database file ( https://sqlite.org). SQLite is a database software that allows to conduct SQL queries on local files. It is listed among the *recommended formats for datasets* of the Library of Congress, which lists data formats that focus on preservation and long-term access ( LoC, 2024). Given that CLDF makes use of the CSVW specification for tabular data on the web ( https://csvw.org, Gower, 2021) and represents all data with the help of CSV tables with additional metadata in JSON, any valid CLDF dataset is fundamentally equivalent to a relational database. Accordingly, the PyCLDF software package ( https://pypi.org/project/pycldf, Forkel *et al.*, 2025b) allows to easily convert any CLDF dataset to SQLite. Instead of accessing individual tables from Lexibank in the form of the original CSV files, we can design SQL queries that solve particular problems and apply them to the SQLite representation of Lexibank data. The advantage of using SQL queries compared to designing search commands on CSV files is a great gain in execution speed. While it may take several minutes on ordinary computers to load the CSV files with Python, not to speak of loading the data with the help of the dedicated PyCLDF package, an SQLite query is usually finished after seconds. Another advantage of SQL queries is that the queries themselves can be easily shared and extended. All that users need to conduct them is a valid installation of SQLite – which is available on most machines.

In order to illustrate the potential of pre-computed features along with the SQLite representation of Lexibank 2, we provide a tutorial with a set of SQLite queries that can be easily used to explore the data in Lexibank in various ways. While these initial queries are only illustrative in nature, we expect that it will be easy to expand them in the future. The three illustrative queries that we offer focus on (1) the automated detection of words that are similar in pronunciation and similar in meaning, (2) the automated inference of cross-linguistic colexifications from the data, and (3) the automated inference of partial colexification patterns. The full tutorial on different queries is published on Codeberg ( https://codeberg.org/lexibank/lexibank-queries/src/tag/v0.1).

***Query Lexibank 2 for similar word forms.*** Our first set of queries retrieves all those word forms in Lexibank 2 that are similar in form and meaning. Similarity in form is measured with the help of *matching consonant classes*, as provided as part of the pre-computed features that extend the regular wordlist aggregated from the individual datasets. Similarity in meaning is measured in a binary fashion by treating all those concepts as similar that are linked to the same central concept and therefore occur in the same semantic community that was inferred for CLICS ^3^. Our queries can be applied in two ways. First, users can define an initial sound class sequence and an initial concept and then search for all word forms in Lexibank 2 that match the first two sound classes and the central concepts. Second, we can compute entire cognate sets within and across language families.

**Figure 2.**
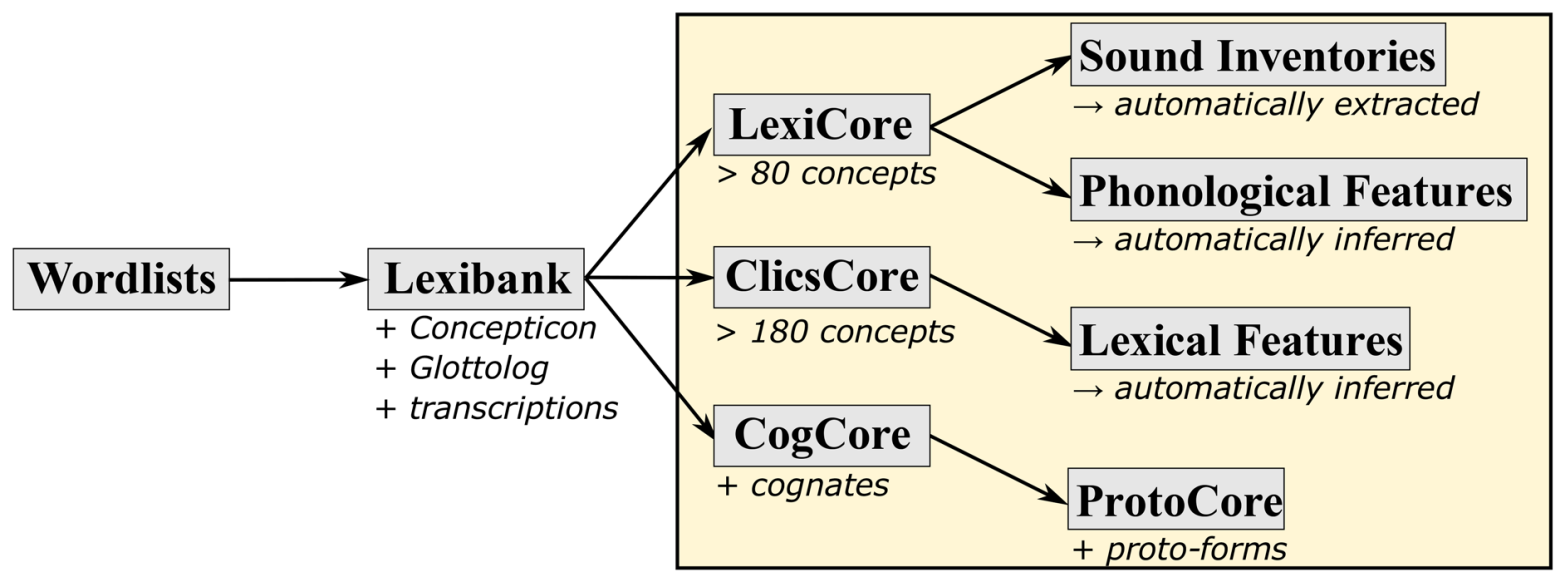
Criteria for the assignment of individual wordlists for individual language varieties to the basic collections of Lexibank 2.

***Query Lexibank 2 for cross-linguistic colexifications.*** A second example for a fast access of the Lexibank data is the fast retrieval of colexifications. While the CLICS ^3^ database ( Rzymski *et al.*, 2020) offers a computation of most of the colexifications found in the first version of Lexibank ( List *et al.*, 2022), it does not cover all concepts present in the data due to a high threshold of concepts for inclusion in ClicsCore (>180, see Figure 2). Through a simple query, we can retrieve cases of colexifications in the dataset for two specific concepts. One can easily add different concepts to the query, with the only requirement being that they are covered by the Concepticon reference catalog and properly linked to it ( List *et al.*, 2025). The output provided is a table of all languages in which the colexification in question can be found along with the word forms in the respective languages.

***Query Lexibank 2 for partial colexifications.*** In contrast to full colexifications that refer to those cases where one word form expresses two or more concepts in the same language, partial colexifications refer to those cases where the word forms expressing two or more different concepts share a certain amount of common lexical material. Partial affix colexifications have so far only been calculated on a small subset of the Lexibank data (a segmented form of the Intercontinental Dictionary Series, https://ids.clld.org, Key & Comrie, 2016b, see List, 2023a). Treating word forms in phonetic transcriptions as sound sequences, List (2023a) – inspired by Urban (2011) – suggests to compute *affix colexifications* from multilingual wordlists by searching for those cases where one word recurs in another word form within the same language variety. The resulting relations (one word being part of another word) can be handled with the help of a directed graph, and statistics similar to the ones computed for full colexifications can be derived, counting, for example, the number of language families in which a certain partial colexification can be identified.

While List (2023a) presents a new method that can efficiently search for partial affix colexifications, we realized that the same result can likewise be achieved with the help of a dedicated query on the Lexibank 2 database. This query iterates over all word pairs that express different concepts within the same language variety and then checks for each word pair if one recurs at the beginning or the end of the other word pair. While List (2023a) correctly points out that this strategy takes a lot of time when running it in Python, it turned out that the computation takes much less time when using the SQLite representation of CLDF. In addition, we can extend the query and not only search for matching substrings in the phonetic entries provided by Lexibank 2, but also apply the same procedure to the sound classes that Lexibank 2 provides as pre-computed features.

## Dataset validation

### Benefits of standardization

The standardization provided by CLDF adds several quality checks to the data in Lexibank 2. Overall, the updated version offers more data in better quality, and thus offers new possibilities for cross-linguistic comparative research. In addition to the aforementioned increase in data points, we fixed errors in Glottocodes, duplicated data points, and incorrect naming of language families. Many of those cases have been brought to our attention by the community of Lexibank users, for which we are very grateful. Furthermore, we have implemented three explicit checks that highlight the benefits of standardization.

The first implemented check is to compare all converted segments against CLTS ( List *et al.*, 2024) to confirm that all segments are represented by standardized symbols. In this way, we avoid the typical pitfalls of Unicode and phonetic transcriptions from which many linguistic datasets suffer ( Moran & Cysouw, 2018). In different linguistic traditions, identical phone qualities might be transcribed with many different graphemes. Similarly, the same grapheme might represent different phonetic qualities. Using the CLTS mapping through orthography profiles, we ensure the comparability that is needed for computer-assisted studies across large datasets such as Lexibank.

When preparing the data for Lexibank 2, we have detected an additional problem that had been overseen in previous Lexibank versions. In order to enhance the phonetic transcriptions, we now compute statistics on consonant clusters, based on the assumption that all clusters in an individual dataset should be roughly checked by experts, paying particular attention to clusters with more than three consonants. This second check allowed us to enhance the individual orthography profiles that we use to convert the original orthographies to phonetic transcriptions in many cases. This check has been run for all individual source datasets of Lexibank 2. Borderline cases, where up to ten consonants would show up in a sequence without a vowel often turned out to go back to the handling of the grapheme <y>, which is often used to represent the glide [j], following a specific transcription practice frequently used in African and South American linguistics, or <ɤ> mapped to [ɣ] (fricative) instead of [ɤ] (vowel). Having identified these problems, we went back to all initial datasets in order to correct these problems.

We applied a third quality check that was an active search for unwanted duplicates in datasets. Duplicates here refer to those cases where one language has two or more identical word forms for the same concept. While one might expect that these cases should be low in general, we detected several thousand duplicates across the 134 datasets and later corrected the errors on the basis of individual datasets as well as by modifying the aggregation procedure. When aggregating Lexibank 2 data, a list of duplicates entries is created automatically and written to a file that is part of the release of the data. Identical entries themselves are not included into the aggregated wordlist.

### Comparing Lexibank 1 and Lexibank 2

We provide a quick comparison between Lexibank 2 and the comparative wordlist shared as part of the previous version of Lexibank in Table 1. From these major characteristics, including the number of language varieties (all doculects from which individual wordlists are populated, including both dialect varieties of the same language and multiple sources for the same language), the number of languages (represented by the number of distinct Glottocodes in both wordlists), and the number of word forms, it can be easily seen that Lexibank 2 drastically expands upon the scope of Lexibank 1.

**Table 1.** Comparison of Lexibank 1 and Lexibank 2.

Criterion	Lexibank 1	Lexibank 2
Datasets	76	134
Varieties	2 028	5 477
Languages	2 028	3 107
Families	192	207
Concepts	3 033	3 205
Words	709 467	1 734 794
Duplicates	12 153	34

With respect to the differences regarding the number of datasets (76 in Lexibank 1 vs. 134 in Lexibank 2), it has to be noted that these refer to the *active datasets*, i.e., datasets that contribute one or more individual wordlists to the comparative wordlist aggregated from the CLDF datasets. Here, we can see that although Lexibank 1 sources data from *more* than 76 CLDF datasets (the wordlist data tries to aggregate from 98 CLDF datasets), there are as many as 22 datasets that are considered but fail to meet the criteria for inclusion into the final wordlist collection. When aggregating data for Lexibank 2, we have made sure that we only include those CLDF datasets into our collection that provide individual wordlists that meet the basic criterion for inclusion, providing at least 80 distinct concepts with phonetic transcriptions. In the table, we have also listed the number of duplicates that we could infer from both datasets when querying the SQLite version of both comparative wordlists. This shows that our targeted efforts aiming at identifying and removing duplicates have been quite successful. We will likewise address the remaining 34 in future versions of Lexibank.

Lexibank 2 ist not only based on adding more data to the existing data in Lexibank. In addition, we have also removed eight datasets in the new version, given that they failed to meet our criterion of providing clear phonetic transcriptions for all entries. The tutorial accompanying this study shows how these datasets can be identified and provides some additional information regarding the reasons that led to their exclusion.

### Queries on pre-computed features

Our queries on pre-computed features allow to search for similar word forms, colexifications, and partial affix colexifications in Lexibank 2. While these queries are useful to explore the data in various ways, they also offer us the possibility to validate the data by checking if the results of the queries correspond to our expectations.

***Similar word forms.*** The first of our queries for similar word forms takes as input a sequence of sound classes and a concept and then searches through all word forms in Lexibank 2 in order to identify words that resemble the word phonetically and semantically. This procedure should not be confused with a valid method for the detection of cognate words (see List *et al.*, 2018 on the latter), but rather as a quick way to allow scholars to get an overview on potential candidates for etymologies, borrowings, or coincidentally similar words in the languages of the world. As an example, we ran a query that searches for (1) words that contain Dolgopolsky’s sound classes t and r as the first two consonant classes, expressing (2) concepts that occur in the same community as wood in CLICS ^3^, and recurring in (3) languages that form part of the Indo-European family. The query yields a total of 162 matches, covering the 32 different concepts in Table 2. Of these entries (all provided in the accompanying online tutorial), not all words are truly cognate. What we can see instead is that the method picks up several true cognates, usually within the same concept, while cognates attested across two or more concepts are rather rare.

**Table 2.** Concepts along with the occurrences in the sample query searching for words in which the first two consonant classes are t and r and the concepts occur in the community with wood as central concept in CLICS ^3^.

Concept	Occurrences
BEAM	5
BEESWAX	3
CANDLE	2
CHISEL	9
CLUB	1
CONCEPT	1
COURT	3
CUSTOM	1
DOORPOST	7
EARWAX	3
FIREWOOD	3
FOOTPRINT	5
FOREST	1
HELL	1
LAW	1
MANNER	6
MAST	2
MATCH	1
NEEDLE (FOR SEWING)	2
PATH	7
PATH OR ROAD	4
POST	4
RAFTER	1
RIDGEPOLE	1
ROAD	7
STICK	3
STREET	1
TORCH OR LAMP	3
TREE	38
TREE STUMP	2
TREE TRUNK	15
WOOD	18

This shows that – as we have mentioned before – the query should not be confused with a simple cognate detection method, but rather taken as a way to broadly search a dataset for words that exhibit rough similarities in form and meaning. When restricting the number of concepts further, however, we can usually find clusters of words that mostly *are* cognate. As an example, consider the word forms in Table 3, which result from the same query but have been filtered for the concepts tree and wood, respectively. Here, we find the typical reflexes of Proto-Indo-European *doru- ‘tree / wood’ that are prominent both in Slavic and Germanic languages.

**Table 3.** Restricting the broad query for words with initial consonant classes t and r to the concepts tree and wood (showing only five matches for each concepts).

Language	Concept	Form
Belarusian	tree	d r ε v a
Bulgarian	tree	d ǝ r v o
Danish	tree	t ʁ εː Ɂ
English	tree	t r iː
Macedonian	tree	d ǝ ɾ v ɔ
Old Church Slavonic	wood	d r u v a
Russian	wood	d r ^j^ ɪ v ^j^ ɪ s ^j^ i n ǝ
Slovak	wood	d r ε v ɔ
Swedish	wood	t r εː
Ukrainian	wood	d r ɔ w ɑ

From this experimental query on similar words in Indo-European, we can learn two lessons. On the one hand, we find that the consonant class matching approach by Dolgopolsky (1964), although pretty simple in nature, works sufficiently well and also sufficiently fast to query large databases such as Lexibank 2 for similar word forms. On the other hand, however, we can see that relaxing semantics, searching for semantically similar rather than semantically identical words, yields large amounts of unrelated words. This shows that any long-range approaches, trying to prove deep genealogical relationships that predate the time depth of the families established by traditional methodology, should be taken with great care, specifically when they relax the role that semantics plays in determining etymologically related words (see Ratcliffe, 2012 for similar arguments on this matter, and Blum *et al.*, 2024a for verifying such cognate sets through colexifications).

***Cross-linguistic colexifications.*** Lexibank 2 continues previous Lexibank versions in inferring several cross-linguistic colexifications as lexical language features and sharing them in the form of a dedicated CLDF dataset published as integral part of the repository. Given that cross-linguistic colexifications on a large scale can be easily inspected through the CLICS database, computing additional colexifications through Lexibank 2 may seem unnecessary, especially since the most recent version of CLICS – CLICS ^4^ – now also models colexifications as lexical language features ( Tjuka *et al.*, 2025). However, since the major idea behind CLICS is to assemble large-scale colexification networks that can then be inspected manually and automatically, it may still be useful to be able to check for individual colexification patterns, if they *are* reflected in Lexibank 2, and if so, which word forms and which languages exhibit them. In our sample query that illustrates how individual colexifications can be queried, we search for potential colexifications of sun and moon. While these may seem to be extremely rare at first sight, it turns out that they are reflected in many different language varieties from several different language families in South America. Table 4 shows the results of the query (with most examples coming from the databases of Chacon, 2017 and Blum *et al.*, 2024b). As can be seen from the individual forms that exhibit the colexification across the six different language families, words inside the same language family all seem to go back to the same root, while no commonality between forms across language families can be identified. This shows that this colexification pattern, as distinct as it may seem, seems to have originated at an earlier point in time in most language families. One may further speculate that the areal frequency of the correlation points back to ancient contact situations.

**Table 4.** Colexification of sun and moon in Lexibank inferred with the query for cross-linguistic colexifications.

Language	Family	Form
Giacone	Arawakan	k eː r i
Bora	Boran	n ɯ Ɂ p a
Mirana	Boran	n ɯ Ɂ p a
Muinane	Boran	n ɨ Ɂ ɨ b a
Guahibo	Guahiboan	h u a m e t o
Guayabero	Guahiboan	h u i m t
Playero	Guahiboan	h u a m e t o
Nukak	Kakua-Nukak	w i d Ɂ
Matses	Pano-Tacanan	u ʂ ɨ
Barasana	Tucanoan	b ũ h $\tilde{\text{i}}$ h ũ
Cubeo	Tucanoan	a w i a
Desano	Tucanoan	a b e
Piratapuyo	Tucanoan	a s $\tilde{ɨ}$
Siriano	Tucanoan	a b e

***Partial affix colexifications.*** Thanks to the SQLite representation of the data in Lexibank 2, it is straightforward to compute partial affix colexifications for a much larger dataset now. Running our dedicated query, we receive as many as 360,649 individual instances of partial colexifications from all datasets in the sample. As shown already in List (2023a), this exceeds the amount of full colexifications that one typically receives largely and also makes clear why it is so important to filter these kinds of colexifications rigorously, retaining only those that recur across several language families. That the method nevertheless retrieves valid and interesting results, can be seen when checking only the 15 most frequently recurring patterns of affix colexifications, which are shown in Table 5.

**Table 5.** Most frequent partial affix colexifications in Lexibank 2.

#	Source	Target	Fams.
1	TEN	ELEVEN	81
2	TEN	TWELVE	80
3	TWO	TWELVE	75
4	ONE	ELEVEN	74
5	FOOT	TOE	58
6	TREE	BARK	56
7	HAND	FINGER	49
8	TWO	SEVEN	48
9	FOUR	NINE	47
10	WOMAN	GIRL	47
11	BREAST	NIPPLE	45
12	MOUTH	LIP	45
13	SKIN	BARK	45
14	TWO	TWENTY	45
15	TEN	TWENTY	42

As a huge advantage of the pre-computed features in Lexibank 2, the investigation of partial colexifications in this form is not limited to the phonetic sequences, but can also be applied to the sound class strings. While this will increase the noise even further, it may help to capture certain cases of allophony in word families that one would not be able to observe when sticking to phonetic transcriptions. Running the modified query on Lexibank 2, using Dolgopolsky’s consonant classes as the major model for sound classes results in a huge increase in observed affix colexification pairs, with 7,930,552 instances. However, when restricting the comparison only to the most frequently recurring 15 colexifications, as shown in Table 6, we find a picture much similar to the one that we have observed in Table 5.

**Table 6.** Most frequent partial affix colexifications in Lexibank 2 when computing partial affix colexifications from sound class strings.

#	Source	Target	Fams.
1	TEN	ELEVEN	91
2	TEN	TWELVE	89
3	ONE	ELEVEN	86
4	TWO	TWELVE	81
5	FOOT	TOE	64
6	TREE	BARK	63
7	WOMAN	GIRL	55
8	HAND	FINGER	54
9	THREE	EIGHT	53
10	FOUR	NINE	50
11	SKIN	BARK	50
12	TWO	SEVEN	50
13	TWO	TWENTY	50
14	WHAT	WHY	50
15	MAN	BOY	49

**Table 7.** Table of all datasets, their number of concepts, the number of concepts mapped to Concepticon, as well as the source of the individual datasets.

Dataset	Concepts	Concepticon	Source
aaleykusunda	230	230	Aaley & Bodt (2019)
abrahammonpa	306	304	Abraham *et al*. (2005)
abvdoceanic	191	191	Greenhill *et al*. (2008)
abvdphilippines	210	210	Greenhill *et al*. (2008)
allenbai	499	499	Allen (2007)
backstromnorthernpakistan	1233	224	Backstrom & Radloff (1992)
baf2	347	335	Hantgan *et al*. (2022)
bantubvd	430	415	Greenhill & Gray (2015)
barlowkilliantomoip	996	623	Barlow & Killian (2023)
barlowlote	551	443	Barlow (2024)
beidasinitic	905	738	Dàxué (1964)
berrywestpapuan	195	195	Berry & Berry (1987); Kamholz *et al*. (2024)
birchallchapacuran	125	125	Birchall *et al*. (2016)
blumpanotacana	501	501	Blum *et al*. (2024b)
blustaustronesian	210	210	Greenhill *et al*. (2008)
bodtkhobwa	662	542	Bodt & List (2022)
bowernpny	344	341	Bowern & Atkinson (2012)
bremerberta	200	183	Bremer (2016)
cals	184	184	Mennecier *et al*. (2016)
carvalhopurus	205	173	de Carvalho (2021)
castrosui	592	518	Castro (2011)
castroyi	540	532	Castro *et al*. (2010)
castrozhuang	510	490	Castro & Hansen (2010)
chacolanguages	323	223	Brid *et al*. (2022)
chaconarawakan	102	100	Chacon (2017)
chaconbaniwa	243	233	Chacon *et al*. (2019)
chaconcolumbian	128	125	Chacon (2017)
chaconnorthwestarawakan	94	94	Chacon (2022)
chacontukanoan	142	127	Chacon (2014)
chenhmongmien	883	799	Chén (2012)
chindialectsurvey	460	451	Language & Social Development Organization (2019)
chingelong	99	98	Chin (2015)
clarkkimmun	757	636	Clark (2008)
constenlachibchan	110	110	Constenla Umaña (2005)
crossandean	150	150	Blum *et al*. (2023)
davletshinaztecan	100	100	Davletshin (2012)
deepadungpalaung	100	100	Deepadung *et al*. (2015)
dhakalsouthwesttibetic	243	240	Dhakal *et al*. (2024)
dravlex	100	100	Kolipakam *et al*. (2018)
dunnaslian	146	146	Dunn *et al*. (2013)
dunnielex	207	207	Dunn (2012)
duonglachi	100	100	Duong *et al*. (2020)
felekesemitic	150	139	Feleke (2021)
galuciotupi	100	100	Galucio *et al*. (2015)
gaotb	100	100	Gao (2020)
gerarditupi	244	242	Gerardi *et al*. (2021)
gravinachadic	717	716	Gravina (2014)
grollemundbantu	100	100	Grollemund *et al*. (2015)
halenepal	997	732	Hale (1973)
hantganbangime	300	300	Hantgan & List (2022)
hattorijaponic	200	200	Hattori (1973)
heathdogon	944	880	Moran *et al*. (2016)
houchinese	140	139	Hóu (2004)
hubercolumbian	366	347	Huber & Reed (1992)
huntergatherer	744	408	Bowern *et al*. (2021)
idssegmented	1310	1308	Key & Comrie (2016a)
iecor	170	170	Heggarty *et al*. (2024)
ivanisuansu	250	250	Ivani (2019)
johanssonsoundsymbolic	344	284	Johansson *et al*. (2020)
joophonosemantic	100	100	Joo (2019)
kesslersignificance	200	200	Kessler (2001)
keypano	1310	1308	Miller & List (2024)
kleinewillinghoeferbikwinjen	119	117	Kleinewillinghöfer (2015)
kochtukanoan	804	445	Koch-Grünberg (1914)
kraftchadic	433	429	Kraft (1981)
lairgyalrong	291	291	Lai & List (2023)
lamanisoic	301	283	Lama (2012)
leeainu	199	199	Lee & Hasegawa (2013)
leecaijia	234	234	Lee (2022)
leejaponic	210	210	Lee & Hasegawa (2011)
leekoreanic	246	246	Lee (2015)
lieberherrkhobwa	100	100	Lieberherr & Bodt (2017)
lindseyende	1129	1128	Lindsey (2019)
lionnetyotonahua	364	292	Lionnet (1985)
listsamplesize	550	549	List (2014a)
liunewari	180	180	Liu *et al*. (2023)
liusinitic	203	202	Liú *et al*. (2007)
luangthongkumkaren	341	249	Luangthongkum (2019)
mannburmish	391	388	Mann (1998)
marrisonnaga	884	827	Marrison (1967)
mcelhanonhuon	140	140	McElhanon (1967)
mitterhoferbena	355	336	Mitterhofer (2013)
mixtecansubgrouping	240	204	Auderset *et al*. (2023)
naganorgyalrongic	1256	900	Nagano & Prins (2013)
nagarajakhasian	200	200	Nagaraja *et al*. (2013)
ndanda	200	200	Douanla Taffre (2021)
northeuralex	1016	954	Dellert *et al*. (2020)
northperulex	200	200	Barrientos *et al*. (Forthcoming)
oskolskayatungusic	254	254	Oskolskaya *et al*. (2022)
othanieljen	300	285	Othaniel (2017)
papuanvoices	215	215	Sawaki *et al*. (2020)
peirosaustroasiatic	100	100	Peiros (1998)
pharaocoracholaztecan	100	100	Hansen (2020)
polyglottaafricana	319	263	Koelle (1854)
ratcliffearabic	100	100	Ratcliffe (2021)
robbeetstriangulation	253	253	Robbeets *et al*. (2021)
robinsonap	398	392	Robinson & Holton (2012)
saenkoromance	110	110	Saenko (2015)
sagartst	250	250	Sagart *et al*. (2019)
savelyevturkic	254	254	Savelyev & Robbeets (2020)
sawkatokaleya	201	199	Sawka *et al*. (2019)
seifartecheverriboran	408	351	Seifart & Echeverri (2015)
servamalagasy	207	207	Serva & Pasquini (2020)
sidwellbahnaric	200	200	Sidwell (2015)
sidwellvietic	116	116	Sidwell & Alves (2021)
simsrma	233	190	Sims (2020)
sohartmannchin	280	279	So-Hartmann (1988)
spagnolmaltese	1277	1275	Spagnol *et al*. (2024)
starostinkaren	110	110	Starostin & Krylov (2011)
starostinpie	110	110	Starostin (2005)
starostintujia	109	109	Starostin (2013)
suntb	1004	931	Sūn (1991)
syrjaenenuralic	173	173	Syrjänen *et al*. (2013)
tls	1052	650	Nurse & Phillipson (1975)
tolmiebritishcolumbia	210	200	Tolmie & Dawson (1884)
transnewguineaorg	1176	888	Greenhill (2015)
tryonsolomon	323	313	Tryon & Hackman (1983)
tuled	447	439	Ferraz Gerardi *et al*. (2022)
utoaztecan	121	121	Greenhill *et al*. (2023)
vanuatuvoices	426	426	Takau *et al*. (2024)
visserkalamang	855	854	Visser (2021)
walkerarawakan	100	100	Walker & Ribeiro (2011)
walworthpolynesian	210	210	Walworth (2018)
wangbai	458	404	Wang (2004)
wheelerutoaztecan	102	101	Wheeler & Whiteley (2015)
wichmannmixezoquean	110	110	Wichmann *et al*. (2011)
wold	1814	1458	Haspelmath & Tadmor (2009)
yanglalo	1000	877	Yang (2011)
yangyi	1001	877	Yang (2022)
yuchinese	1307	1305	Yu & Wang (2021)
zgraggenmadang	333	310	Z’graggen (1980)
zhaobai	201	200	Zhao (2006)
zhivlovobugrian	110	110	Zhivlov (2011)
zhoubizic	346	301	Zhou (2020)

All in all, this shows that it may be worthwhile to further investigate partial affix colexifications computed from sound class representations. Apart from comparing the resulting networks or the individually inferred colexifications, it may also be interesting to see to which degree the resulting concept embeddings computed from sound class colexifications differ from concept embeddings computed from phonetic transcriptions ( Rubehn & List, 2025).

### Limitations of Lexibank queries

Querying the Lexibank collection for various purposes offers scholars the possibility to dive into the resource and explore cross-linguistic patterns in various ways. Given the sheer size of Lexibank as well as the fact that it is impossible to verify and double-check every single data point that the resource offers, the results of these queries should, however, be taken with a certain care. Past research has shown that even manually curated datasets often show remarkable differences, even when coding for the same language varieties ( Anderson *et al.*, 2023) As a result, it seems even more important to carry out additional tests to verify that the data retrieved from Lexibank is useful for a given analysis. In this context, what is considered as useful and what not, depends -- obviously -- from the analysis at hand. For example, as List (2018) has shown, rudimentary patterns of consonant clusters in the world's languages can be easily inferred from Lexibank and similar data collections. Whether a concrete analysis of these results, as presented by Yin *et al.* (2023), however, is meaningful and stands rigorous tests by follow-up studies, is different question that can only be answered in future research. With Lexibank we hope to facilitate linguists' access to cross-linguistic data in the domain of the lexicon. This does not mean, of course, that the collection also facilitates the interpretation of the results obtained with the data. We hope that future engagement with Lexibank will help in finding out where the data may be useful and where its limitations lie.

### Beyond Lexibank

Our work on Lexibank has shown the benefits of standardizing data in order to allow for large-scale data aggregation. However, given that the purposes of individual data collections may well differ in practice, the work on Lexibank has also shown that a single repository cannot accommodate all needs at once. What we often need instead are targeted collections that concentrate on particular aspects of cross-linguistic data, thus offering targeted solutions for detailed problems. Examples are the fourth installment of the *Database of Cross-Linguistic Colexifications* ( Tjuka *et al.*, 2025), initial efforts to derive benchmark datasets from the data in Lexibank 2 ( Häuser & List, 2025), new approaches to deriving correspondence patterns from datasets with alignments and cognate annotation ( Blum & List, 2023), merging and analysis of family-specific datasets Blum *et al.* (2024a), as well as targeted web-based applications that allow for a targeted inspection of retro-standardized data ( Forkel & List, 2024). In all these cases, the typical analyses that scholars desire to carry out largely exceed the needs of what a single data collection such as Lexibank 2 can offer. As a result, we do not only want to encourage colleagues interested in Lexibank 2 to test out different ways to query the data in its aggregated form, but also hope that new, particularly targeted collections might emerge from the individual datasets that we standardized for Lexibank 2.

## Ethics and consent

Ethical approval and consent were not required.

## Data Availability

Table 7 presents an overview of the individual datasets. The data is curated on GitHub ( https://github.com/lexibank/lexibank-analysed/tree/v2.1) and released on Zenodo ( Blum *et al.* (2025a), https://doi.org/10.5281/zenodo.15194559). The queries are published in a tutorial on Codeberg ( https://codeberg.org/lexibank/lexibank-queries/src/tag/v0.1). Data are available under the terms of the Creative Commons Attribution 4.0 International license (CC-BY 4.0).
