# Supplementary material for: Lexibank 2: pre-computed features for large-scale lexical data
Source: Open Res Eur. 2025 Jun 23;5:126. Originally published 2025 May 9. [Version 2] doi: 10.12688/openreseurope.20216.2 (PMC12134731; doi:10.12688/openreseurope.20216.2)
Supplement: Peer review history

# Reviewer report: Reviewer response for version 2

**DOI:** 10.21956/openreseurope.22414.r55975

**Published:** 2025-07-02

**Authors:** César Espin-Riofrio

**Affiliations:** 1 Universidad de Guayaquil, Guayaquil, Ecuador

No further comments.

Are sufficient details of methods and materials provided to allow replication by others?

Yes

Is the rationale for creating the dataset(s) clearly described?

Yes

Are the datasets clearly presented in a useable and accessible format?

Yes

Are the protocols appropriate and is the work technically sound?

Yes

Reviewer Expertise:

Natural Language Processing

I confirm that I have read this submission and believe that I have an appropriate level of expertise to confirm that it is of an acceptable scientific standard.

# Reviewer report: Reviewer response for version 2

**DOI:** 10.21956/openreseurope.22414.r55976

**Published:** 2025-07-02

**Authors:** Chundra Cathcart

**Affiliations:** 1 University of Zurich, Zürich, Switzerland

My main comments have been adequately addressed.

Are sufficient details of methods and materials provided to allow replication by others?

Yes

Is the rationale for creating the dataset(s) clearly described?

Yes

Are the datasets clearly presented in a useable and accessible format?

Yes

Are the protocols appropriate and is the work technically sound?

Yes

Reviewer Expertise:

Historical linguistics, evolutionary linguistics

I confirm that I have read this submission and believe that I have an appropriate level of expertise to confirm that it is of an acceptable scientific standard.

# Reviewer report: Reviewer response for version 2

**DOI:** 10.21956/openreseurope.22414.r55977

**Published:** 2025-06-25

**Authors:** Dan Dediu

**Affiliations:** 1 University of Barcelona, Barcelona, Spain

it seems ok to me, except that I think Figure 1 is lacking a full legend (e.g., there's no description of what the colors and shapes mean)

Are sufficient details of methods and materials provided to allow replication by others?

Yes

Is the rationale for creating the dataset(s) clearly described?

Yes

Are the datasets clearly presented in a useable and accessible format?

Yes

Are the protocols appropriate and is the work technically sound?

Yes

Reviewer Expertise:

Linguistic diversity; computational methods; statistics

I confirm that I have read this submission and believe that I have an appropriate level of expertise to confirm that it is of an acceptable scientific standard.

# Reviewer report: Reviewer response for version 1

**DOI:** 10.21956/openreseurope.21876.r54517

**Published:** 2025-06-03

**Authors:** César Espin-Riofrio

**Affiliations:** 1 Universidad de Guayaquil, Guayaquil, Ecuador

The article clearly describes the standardization challenges in comparative linguistics and how Lexibank 2 addresses them, thereby justifying the expansion of the previous dataset (Lexibank 1).

 The methodology is based on well-established tools such as CLDF, PyLexibank, and CLTS. The use of SQLite for large-scale querying reflects a technically rigorous approach.

 The workflow for data conversion and validation is extensively documented, including the use of specific software packages, the integration with reference catalogs, and the criteria for data inclusion.

 The datasets are publicly available on Zenodo and GitHub. Moreover, the data are provided in the CLDF format, a widely adopted and thoroughly documented standard. Ready-to-use SQL queries, tutorials, and complete source files are also made available.

 Several specific adjustments are recommended. First, it would be advisable to include a more systematic empirical validation of the phonetic and semantic queries, as they are currently presented as exploratory tools without a formal assessment of their performance. Additionally, it is recommended to clarify in greater detail the criteria used to exclude certain datasets, which would help make the curation process more transparent.

 It would also be useful to reinforce the cautions regarding the interpretation of the results, particularly colexifications, to avoid misleading inferences about linguistic relationships. Finally, the inclusion of a brief comparison between versions of Lexibank is suggested, to help users better understand the changes introduced and their impact on the resource’s use.

Are sufficient details of methods and materials provided to allow replication by others?

Yes

Is the rationale for creating the dataset(s) clearly described?

Yes

Are the datasets clearly presented in a useable and accessible format?

Yes

Are the protocols appropriate and is the work technically sound?

Yes

Reviewer Expertise:

Natural Language Processing

I confirm that I have read this submission and believe that I have an appropriate level of expertise to confirm that it is of an acceptable scientific standard.

# Reviewer report: Reviewer response for version 1

**DOI:** 10.21956/openreseurope.21876.r54153

**Published:** 2025-06-03

**Authors:** Chundra Cathcart

**Affiliations:** 1 University of Zurich, Zürich, Switzerland

I think this paper and the release of Lexibank 2 are very timely and I recommend acceptance. Below, I give a précis with some feedback woven in, along with stray comments. The paper proceeds as follows:

 The introduction gives some context on the use of lexical and grammatical databases in historical work. In general there is a great deal more that can be cited in terms of work using cognacy databases to infer phylogenies or carry out phylogenetic comparative methods, but this is also understandably not the focus of the paper. Some details on the historical development of Lexibank are provided as well.

 The section "Materials and methods" describes the structure of Lexibank 2, steps taken to process and normalize the data, and describes the possibility of exploiting the data's relational structure for SQL queries. Data sets in lexibank are present in different collections depending on what features are available. The subsection "Workflow for data curation" details steps involving the removal of certain data sets (specifically, ones that cannot be converted to phonemic transcription —— I would have been curious to know what specific traits characterize resources that cannot be converted to phonemic transcription; this was not entirely clear to me although the excluded resources are listed in the online documentation). This subsection also mentions alternative ways of dealing with languages that are duplicated across datasets, and lists some precomputed phonological and semantic features (e.g., conversion of segments to sound classes, assignment of central concepts, etc.). The subsection "Queries for data exploration" outlines some possible data retrievals that can be undertaken using SQLite.

 The section "Dataset validation" is essentially a Results section describing the outcomes of certain processing steps and SQL queries. The subsection "Benefits of standardization" details some quality checks, including heuristics used to find incorrectly converted segments. The subsection "Queries on pre-computed features" gives the results of three queries (similar word forms, cross-linguistic colexifications, and partial colexifications, the latter of which is challenging given the string matching needed, and best carried out using SQL rather than Python). The first query presents a worked example that involves finding all forms with T and R as C1 and C2 with connection to WOOD, which returns a number of reflexes of PIE *doru in Germanic and Slavic (a subset of which are given in Table 3). It would have been nice to actually use gold standard cognacy judgments (which if I am not mistaken should be available for IE languages) to get a sense of not only the precision but also the recall of this strategy in finding cognates (e.g., do we get hits for the IA, Hellenic, etc., reflexes of this PIE etymon?). And while the two lessons contrasted at the end of this example (p. 9: "From this experimental query...") are well taken (semantic permissiveness coupled with Dolgopolsky's sound class method is often criticized, e.g., https://doi.org/10.1075/jhl.00018.ceo), the wording here is confusing: TREE and WOOD are semantically closer than e.g. EARWAX, but they are still not identical. 

 I have to admit that I found it a bit disorienting to have to refer back to the previous section when reading the results described, but this may just be my personal preference.

 All in all, the steps taken to normalize the data are well described. However, one message that did not always come through clearly in the text pertains to what researchers should be using these precomputed features for —— and what they should *not* be using them for. It is mentioned in passing that we should not uncritically take queries involving Dolgopolsky's method to represent cognates. But I found myself wondering for instance if we could take syllable complexity at face value (it is very easy to automatically extract syllable templates, but different languages may syllabify CVCCV differently) for use in analyses. Or are these features more geared towards some downstream application (e.g., for use in cognate detection)? An analogy is the use of byte-pair encoding in NLP: as a linguistic representation, it is pretty meaningless, but it improves performance in language modeling when used as a tokenization step in preprocessing. Some clearer guidance along these lines would likely be helpful to Lexibank 2 users, and it would not hurt to repeat any cautionary statements at various places in the paper (e.g., the conclusion).

 Other issues:

 Figure 1: I am not a fan of using pie charts to represent multiple variables on a map, as visibility is challenging. I also don't understand why the redundant feature "LexiCore" is included, since all doculects have the value 1. Removing this variable would improve visibility (via e.g. a 50/50 pie chart or color/shape aesthetic parameterization).

 Some URLs are broken: https://codeberg.org/lexibank/lexibank-queries/v0.1 was not found, though https://codeberg.org/lexibank/lexibank-queries worked fine.

 Table 7: weird diacritic in "Zǵraggen (1980)"

 p. 18: "which is often used to represent the glide [i]" -> "glide [j]"?

Are sufficient details of methods and materials provided to allow replication by others?

Yes

Is the rationale for creating the dataset(s) clearly described?

Yes

Are the datasets clearly presented in a useable and accessible format?

Yes

Are the protocols appropriate and is the work technically sound?

Yes

Reviewer Expertise:

Historical linguistics, evolutionary linguistics

I confirm that I have read this submission and believe that I have an appropriate level of expertise to confirm that it is of an acceptable scientific standard.

# Reviewer report: Reviewer response for version 1

**DOI:** 10.21956/openreseurope.21876.r54158

**Published:** 2025-06-03

**Authors:** Dan Dediu

**Affiliations:** 1 University of Barcelona, Barcelona, Spain

I found the article very well written and clear, the examples useful, and there is no question that this is a potentially very useful extension of the previous version of Lexibank.

 So, overall, I have no real comments nor questions, with the exception of something that I personally found a bit unclear in the paper: so, could the differences and similarities between Lexibank and CLICS be made clearer? I think this could be quite important for this new version of Lexibank.

 Minor issues:

 p4: "were be" -> "were"?

 Figure 1: the map is way too busy to make any sense of it, especially for people with abnormal colour perception: I suggest breaking it down in at least 3 different maps (one figure with 3 panels or 3 separate figures), if possible -- that would also get rid of the very busy and hard-to-see circle sectors as well

Are sufficient details of methods and materials provided to allow replication by others?

Yes

Is the rationale for creating the dataset(s) clearly described?

Yes

Are the datasets clearly presented in a useable and accessible format?

Yes

Are the protocols appropriate and is the work technically sound?

Yes

Reviewer Expertise:

Linguistic diversity; computational methods; statistics

I confirm that I have read this submission and believe that I have an appropriate level of expertise to confirm that it is of an acceptable scientific standard.
